# Placental Alterations in Autism Spectrum Disorder: An In Silico Approach to circRNA–miRNA–mRNA Networks

**DOI:** 10.1002/jdn.70064

**Published:** 2025-11-11

**Authors:** Brayan Braz‐Barbosa, Carmem Gottfried, Júlio Santos‐Terra

**Affiliations:** ^1^ Translational Research Group in Autism Spectrum Disorder–GETTEA Universidade Federal do Rio Grande do Sul (UFRGS) Porto Alegre Brazil; ^2^ Department of Biochemistry Universidade Federal do Rio Grande do Sul (UFRGS Porto Alegre Brazil; ^3^ National Institute of Science and Technology in Neuroimmunomodulation–INCT‐NIM Brazil; ^4^ Autism Wellbeing and Research Development (AWARD)–BR‐UK‐CA Initiative

**Keywords:** ASD, bioinformatics, circRNA, circRNA–miRNA–mRNA, placenta

## Abstract

Autism spectrum disorder (ASD) is a neurodevelopmental condition characterized by deficits in social communication and repetitive behaviours with an aetiology involving genetic and environmental risk factors. Placental alterations, such as epigenetic DNA methylation and structural abnormalities, have been associated with ASD. Circular RNA (circRNA), covalently closed and highly stable molecules, play an epigenetic role by sequestering microRNA (miRNA) and modulating messenger RNA (mRNA) translation, forming posttranscriptional networks essential for gene expression. However, there is a lack of evidence in the literature regarding the involvement of circRNA, the placenta and ASD. To address this gap, the study aimed to map the interactions among circRNA, miRNA and mRNA, investigating their relevance to ASD and placental development using bioinformatics tools, such as *circATLAS* and *miRTargetLink* 2.0. The analysis identified 71 circRNA linked to ASD and 30 highly expressed in the placenta, which regulate pathways such as ‘immune response,’ ‘gene transcription,’ and ‘replication,’ and others previously associated with ASD, such as ‘Notch and AKT signalling pathway’. Searches in the *SFARI* database revealed 11 relevant genes in the ASD group, nine in the placenta group and five shared genes (*SRSF11*, *PSMD11*, *NOTCH1*, *CREBBP* and *TBL1X*). Further analysis identified the interaction of the circRNA hsa‐MAN1A2_0008 with miRNA associated with these genes. These findings suggest that highly expressed circRNA in the placenta regulate critical pathways for placental development and ASD aetiology, underscoring their role in linking placental alterations to ASD.

## Graphical abstract

The study identified circRNA–miRNA–mRNA interactions in ASD and placenta, finding 71 ASD‐linked circRNA and 30 highly expressed in the placenta. Five genes (SRSF11, PSMD11, NOTCH1, CREBBP, TBL1X) overlapped, with hsa‐MAN1A2_0008 showing a key regulatory potential, highlighting circRNA as possible mediators between ASD and placental alterations.

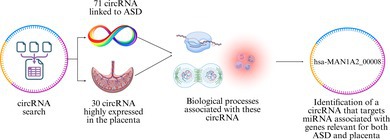

## 1 Introduction

Autism spectrum disorder (ASD) is characterized by persistent deficits in social communication and restricted, repetitive patterns of behaviour, interests or activities (American Psychiatric Association and American Psychiatric Association DSM‐5 Task Force 2013). Over recent decades, the global prevalence of ASD has increased significantly. In the United States, recent estimates suggest that 1 in 36 children under the age of eight is on the spectrum (Maenner 2023). In Brazil, projections from the Brazilian Institute of Geography and Statistics estimate that approximately 2 million people have ASD, representing 1% of the population (IBGE 2023). Although increased awareness and advancements in diagnostic criteria have contributed to this rise, unknown or unclear risk factors continue to represent a significant challenge for ASD research (Zeidan et al. 2022).

The aetiology of ASD is multifactorial, involving a complex interplay between genetic and environmental risk factors (Gottfried et al. 2015). Genetically, alterations in specific loci play a central role in the development of the disorder (Caglayan 2010; Geschwind 2011). Environmentally, multiple factors affecting foetal development during gestational and perinatal periods are implicated, including exposure to teratogenic substances such as valproic acid (VPA) or disruptions in the maternal immune system (Estes and McAllister 2016; Gentile 2014). In addition, epigenetic risk factors, such as DNA methylation and histone modifications, have gained attention for their role in potentially influencing ASD susceptibility during neurodevelopment.

In this context, the placenta—a key component of foetal neurodevelopment—directly influences gene expression and epigenetic processes associated with ASD (Rosenfeld 2021). Alterations in placental DNA methylation, such as those observed in the *CYPE2E1* and *IRS2* genes, as well as structural anomalies, including altered vascular networks, have been linked to an increased risk of developing ASD (Kliman et al. 2021; Zhu et al. 2019).

Noncoding RNA, such as circular RNA (circRNA) and microRNA (miRNA), has gained attention because of its role in posttranscriptional gene regulation and epigenetics. CircRNA is generated through back‐splicing, where a downstream splice donor joins an upstream splice acceptor, forming a covalently closed‐loop structure (Zhang et al. 2016). Additionally, circRNA contains binding sites for miRNA, acting as molecular sponges that sequester these targets, reducing their availability and indirectly modulating messenger RNA (mRNA) translation (Hansen et al. 2015; Memczak et al. 2013). MiRNA, in turn, is a class of noncoding RNA, approximately 21 nucleotides long, found across multiple species, including animals, plants and viruses. It primarily regulates mRNA translation negatively and plays critical roles in neural development and responses to environmental factors (Bartel 2009; Carthew and Sontheimer 2009).

The interaction among circRNA, miRNA and mRNA produces a fundamental regulatory network in posttranscriptional mechanisms, directly influencing gene expression in various biological contexts (Su et al. 2023). This dynamic interaction is pivotal for maintaining cellular homeostasis and coordinating critical biological processes (Saliminejad et al. 2019). Although bioinformatics analyses have identified significant associations with Alzheimer's disease, experimental evidence links circRNA–miRNA–mRNA interactions to early‐onset schizophrenia in children (Huang et al. 2023; Su et al. 2023), the correlation between the interaction networks, placenta and ASD has not yet been made.

Recent research highlights the importance of integrating genetic, epigenetic and transcriptomic data as promising strategies for understanding the mechanisms underlying ASD. Identifying biomarkers, including those in the placenta and noncoding RNA networks, opens new opportunities for early diagnoses and targeted therapeutic interventions, potentially transforming the clinical approach to the disorder (Bahado‐Singh et al. 2022; Saliminejad et al. 2019). Besides that, we focused on the question ‘If a circRNA is altered in ASD, what could be the consequences if the same circRNA were altered during placental development?’ To address this question, the present study conducted an in silico analysis of potential circRNA targets associated with ASD, their interactions with miRNAs and the connections to placental alterations.

## 2 Material and Methods

### 2.1 Literature Screening

**FIGURE 1:**
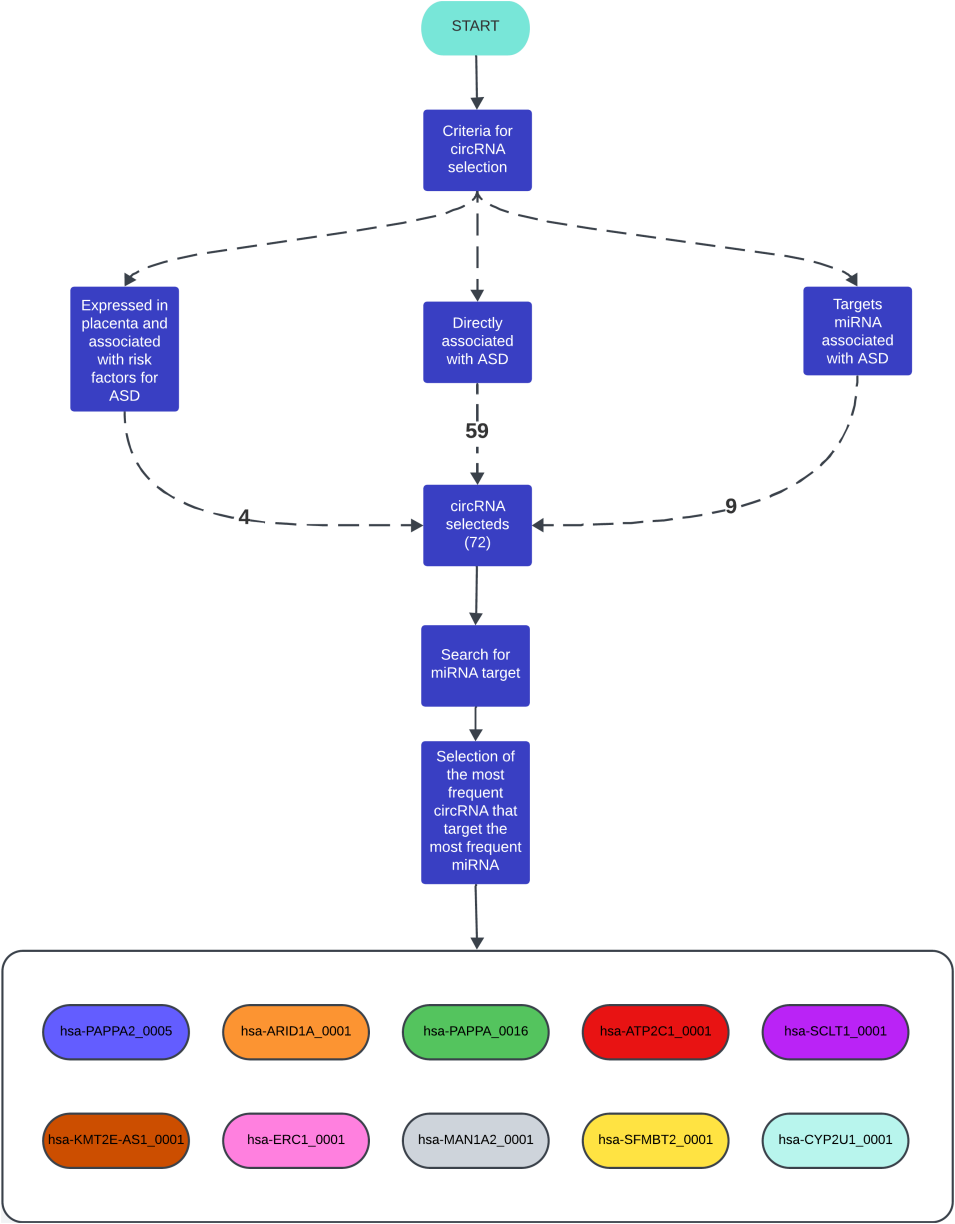
**CircRNA searches for the ASD group.** Fluxogram showing that four circRNA were expressed in the placenta and associated with ASD risk factors; 59 were associated with ASD, and eight circRNA were associated with miRNA described as altered in the ASD, totalizing 71 circRNA. After the selection of miRNA, the reverse search for circRNA elicited 10 circRNA, which are described in the coloured ellipses.

In this study, circRNA was identified through two complementary approaches. First, a literature search was performed in PubMed to retrieve circRNA differentially expressed in ASD. From this search, we identified 59 circRNAs reported in postmortem brain samples from patients with ASD, as well as nine circRNAs described in studies that associated them with miRNA previously implicated in ASD. In addition, 5 circRNAs were identified as being expressed in the placenta and associated with ASD risk factors, based on the circATLAS database. Finally, circATLAS was also used to select the 30 most highly expressed circRNAs in the placenta, as well as to retrieve the predicted miRNA targets of all circRNAs included in the study (Figure 1).

### 2.2 circAtlas

The circRNA identified in the literature screening was submitted separately to circATLAS (Wu et al. 2023) to retrieve their target miRNA. The selected miRNA were compiled and organized to find the most frequent ones. By doing a reverse search, the most frequent miRNA (eight or more for the ASD group and five or more for the placenta group) were used to identify circRNA that most frequently affected these miRNA. Using a reverse‐search workflow, we selected the most frequent miRNAs, defined as those targeted by ≥ 8 circRNAs in the ASD group and ≥ 5 circRNAs in the placenta group, and used them to identify the circRNAs most recurrently targeting these miRNAs. The cutoffs were set at the elbow of the empirical in‐degree distributions and scaled to input size (higher for ASD, lower for placenta). This strategy prioritizes high‐degree hubs while preserving biologically relevant miRNAs, balancing coverage, stringency and interpretability.

### 2.3 miRTargetLink 2.0

The selected miRNA was submitted to *miRTargetLink* 2.0 using the unidirectional search tool in the human database and selecting strong validated targets (Kern et al. 2021). The resulting gene sets were functionally annotated to biological pathways via Reactome/GeneTrail, which relies on overrepresentation analysis (ORA), based on a hypergeometric distribution test that evaluates whether a given pathway contains more submitted genes than would be expected by chance. This test produces a probability score (*p* value), which is further corrected for multiple testing using the Benjamini–Hochberg false discovery rate (FDR) method (< 0.05 as the significance threshold). The background (genome) was defined as the set of *Homo sapiens* in Reactome (version 94; accessed in December 2024); gene identifiers were mapped to HGNC symbols.

### 2.4 SRplot

The main target genes and biological pathways identified in the previous step were input into *SRplot* software to generate a gene ontology (GO) chord graph, connecting the genes to their associated biological pathways. The pathways were identified using a different pattern of colours (Tang et al. 2023). The pathways were differentiated by distinct colour patterns (Tang et al. 2023), and to improve visualization, up to 11 of the most enriched pathways per group were selected.

### 2.5 SFARI

The main target genes were then submitted to the SFARI database to determine whether they had been previously described in experimental or clinical ASD research (Banerjee‐Basu and Packer 2010).

## 3 Results

### 3.1 circRNA in ASD and Placenta

**FIGURE 2:**
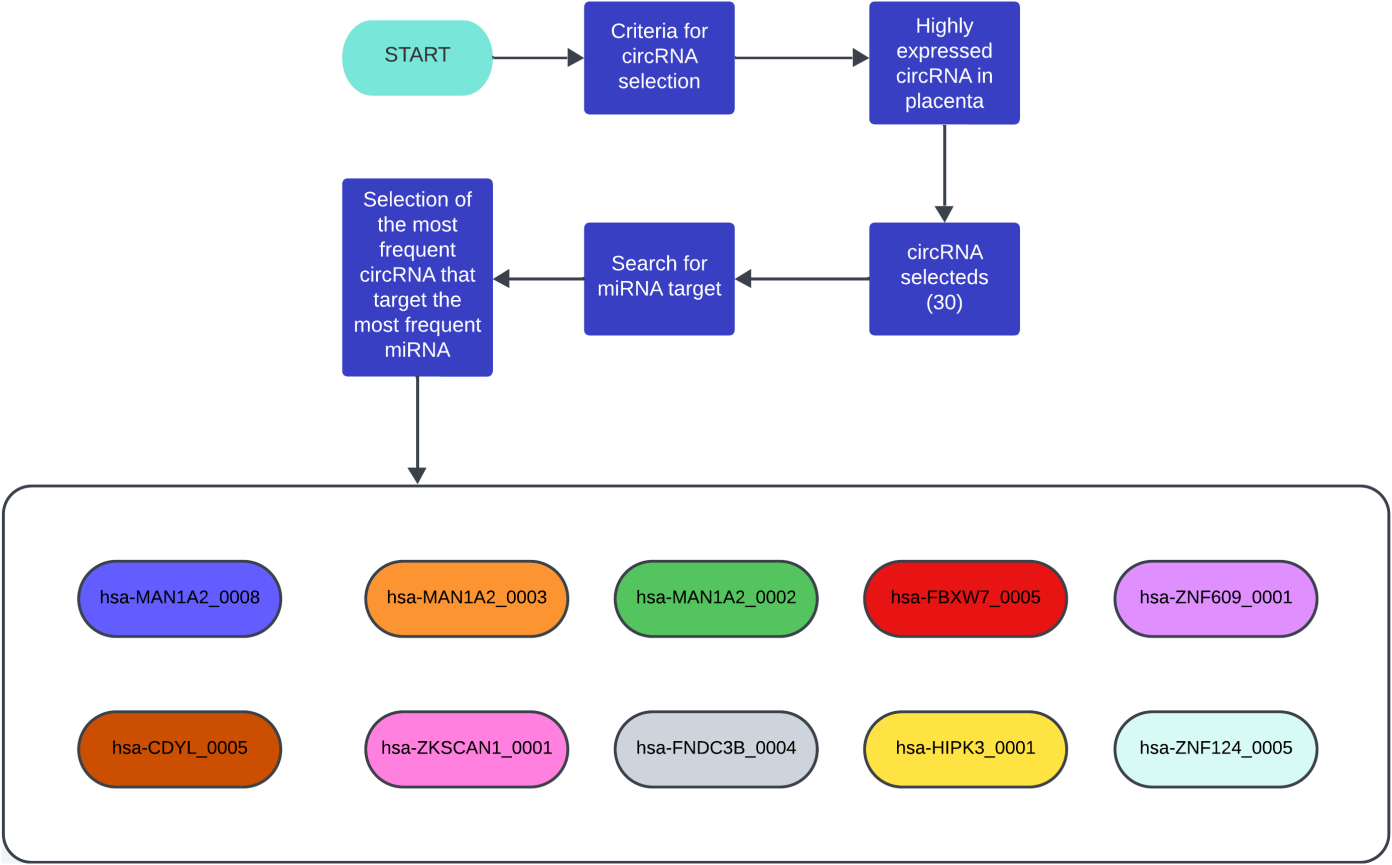
**CircRNA searches for the placenta group.** Fluxogram showing that 30 circRNA were selected using *circAtlas* expression database. After the selection of miRNA, the reverse search for circRNA elicited 10 circRNA, which are described in the coloured ellipses.

The circRNA was divided into two main groups. The first group, referred to as the ASD group, elicited 10 primary circRNA: hsa‐PAPPA2_0005, hsa‐KMT2E‐AS1_0001, hsa‐ARID1A_0001, hsa‐ERC1_0001, hsa‐PAPPA_0016, hsa‐MAN1A2_0001, hsa‐ATP2C1_0001, hsa‐SFMBT2_0001, hsa‐SCLT1_0001 and hsa‐CYP2U1_0001. The second group, referred to as the Placenta group, included 30 circRNA highly expressed in the placenta. The miRNA selection followed by the reverse search revealed 10 main circRNA (hsa‐MAN1A2_0008, hsa‐CDYL_0005, hsa‐MAN1A2_0003, hsa‐ZKSCAN1_0001, hsa‐MAN1A2_0002, hsa‐FNDC3B_0004, hsa‐FBXW7_0005, hsa‐HIPK3_0001, hsa‐ZNF609_0001 and hsa‐ZNF124_0005) (Figure 2).

### 3.2 Selection of the Most Frequent miRNA and Reverse Search for the Respective circRNA

**FIGURE 3:**
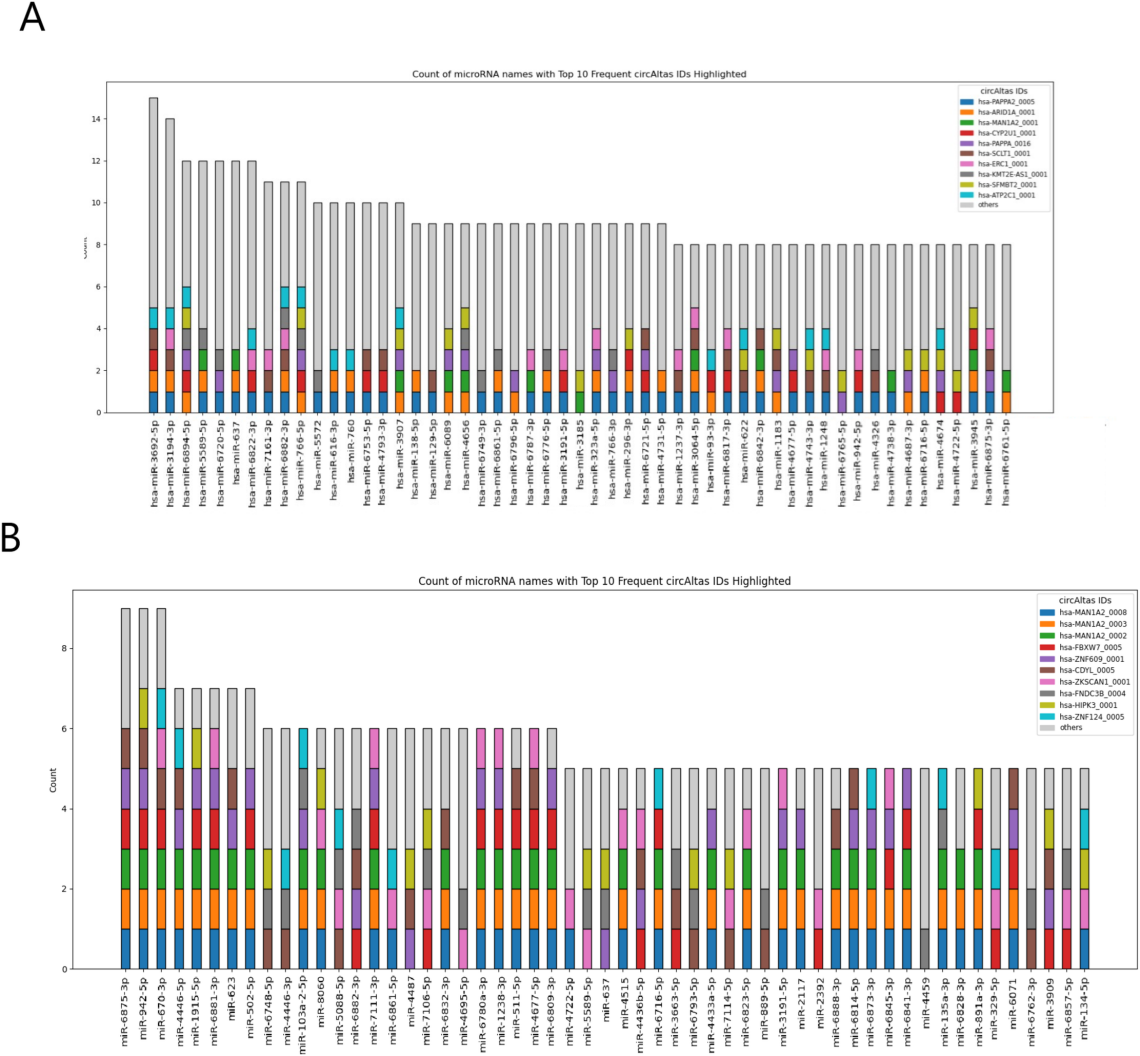
**miRNA x circRNA interaction.** Bar graph showing (A) ASD and (B) association in the placenta group. In the X axis, the most frequent miRNA and in the bars the associated circRNA. The most frequent circRNA are identified by colour.

For both groups, miRNA targets were identified using the circATLAS platform, and the most frequently occurring miRNA was selected. The top 10 circRNA targeting these miRNA were chosen for further analysis. In the ASD group, the most frequent miRNA was targeted by 15 circRNA, while the least frequent was targeted by 8. In the Placenta group, the most frequent miRNA was targeted by 9 circRNA, and the least frequent by 5. These findings are visualized in Figure 3, where circRNA are colour‐coded and organized by their respective miRNA targets.

### 3.3 Selection of the Most Relevant Biological Pathways Based on the miRNA Output

To identify the most relevant circRNA and associated pathways, we conducted an enrichment analysis. For the ASD group, we analyzed miRNA targeted by at least eight circRNA, while for the placenta group, the threshold was miRNA targeted by at least five circRNA. The mirTargetLink platform was used to identify Reactome pathways enriched by these miRNA. To ensure biological relevance, we focused on pathways enriched by at least 15 genes and prioritized those involved in key biological processes such as ‘transcription,’ ‘replication,’ and ‘immune response’ and pathways previously linked to ASD, including ‘Notch and AKT signalling.’ This approach resulted in the identification of 72 pathways in the ASD group and 70 in the Placenta group (Tables S3 and S4).

**FIGURE 4:**
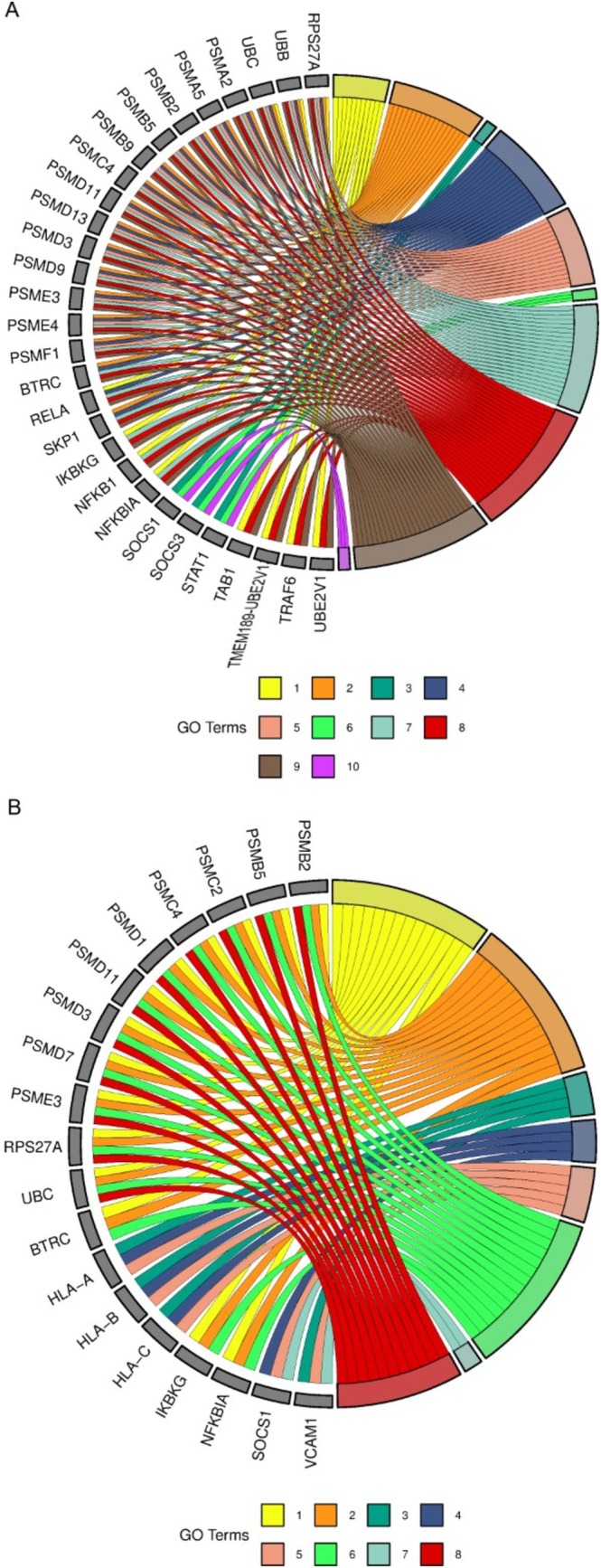
**Immune system pathways.** (A) Enriched immune system pathways for the ASD group. 1) TAK1 activates NF‐kB by phosphorylation and activation of IKKs complex; 2) noncanonical NF‐kB signalling; 3) interferon α/β signalling; 4) dectin‐1–mediated noncanonical NF‐kB signalling; 5) TNFR2 noncanonical NF‐kB pathway; 6) interferon‐γ signalling; 7) activation of NF‐kB in B cells; 8) FCERI‐mediated NF‐kB activation; 9) interleukin‐1 signalling; and 10) interleukin‐4 and interleukin‐13 signalling. (B) enriched immune system pathways for the placenta group. 1) Activation of NF‐kappaB in B cells; 2) FCERI‐mediated NF‐kB activation; 3) immunoregulatory interactions between a lymphoid and a nonlymphoid cell; 4) interferon α/β signalling; 5) interferon‐γ signalling; 6) interleukin‐1 signalling; 7) interleukin‐4 and interleukin‐13 signalling; 8) TNFR2 noncanonical NF‐kB pathway. GO = gene ontology. *P* values were corrected for multiple testing using the Benjamini–Hochberg false discovery rate (FDR), and pathways were considered significant at FDR‐adjusted *p* < 0.05.

**FIGURE 5:**
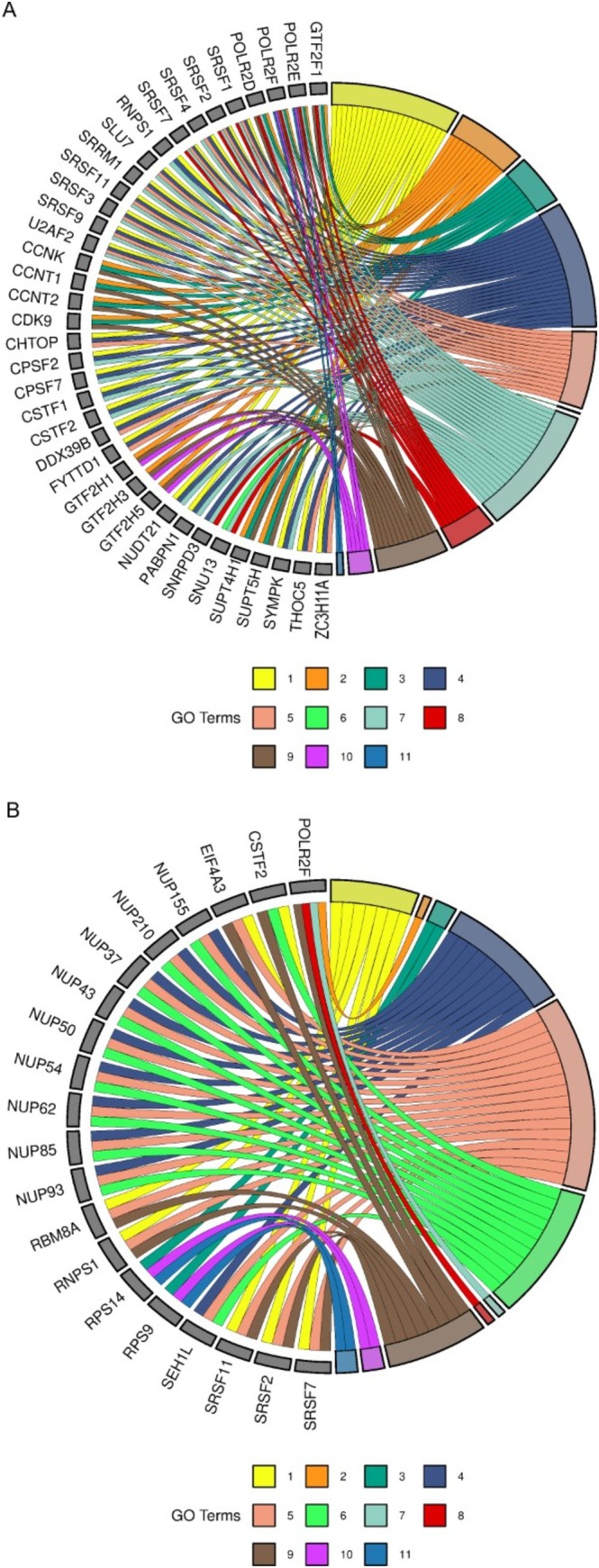
**Enriched transcription pathways**. (A) Enriched transcription pathways for the ASD group: 1) mRNA 3′‐end processing; 2) RNA polymerase II pretranscription events; 3) RNA polymerase II transcribes snRNA genes; 4) RNA polymerase II transcription termination; 5) transport of mature mRNA derived from an intron‐containing transcript; 6) major pathway of rRNA processing in the nucleolus and cytosol; 7) mRNA splicing—major pathway; 8) mRNA splicing—minor pathway; 9) RNA polymerase II transcription elongation; 10) RNA polymerase I transcription initiation; 11) RNA polymerase III abortive and retractive initiation. (B) Enriched transcription pathways for the placenta group: 1) RNA polymerase II transcription termination; 2) RNA polymerase I transcription initiation; 3) rRNA modification in the nucleus and cytosol; 4) SUMOylation of RNA binding proteins; 5) transport of mature mRNA derived from an intron‐containing transcript; 6) tRNA processing in the nucleus; 7) RNA polymerase III abortive and retractive initiation; 8) RNA polymerase II transcribes snRNA genes; 9) mRNA splicing—major pathway; 10) translation initiation complex formation; 11) eukaryotic translation termination. GO = gene ontology. *P* values were corrected for multiple testing using the Benjamini–Hochberg false discovery rate (FDR), and pathways were considered significant at FDR‐adjusted *p* < 0.05.

**FIGURE 6:**
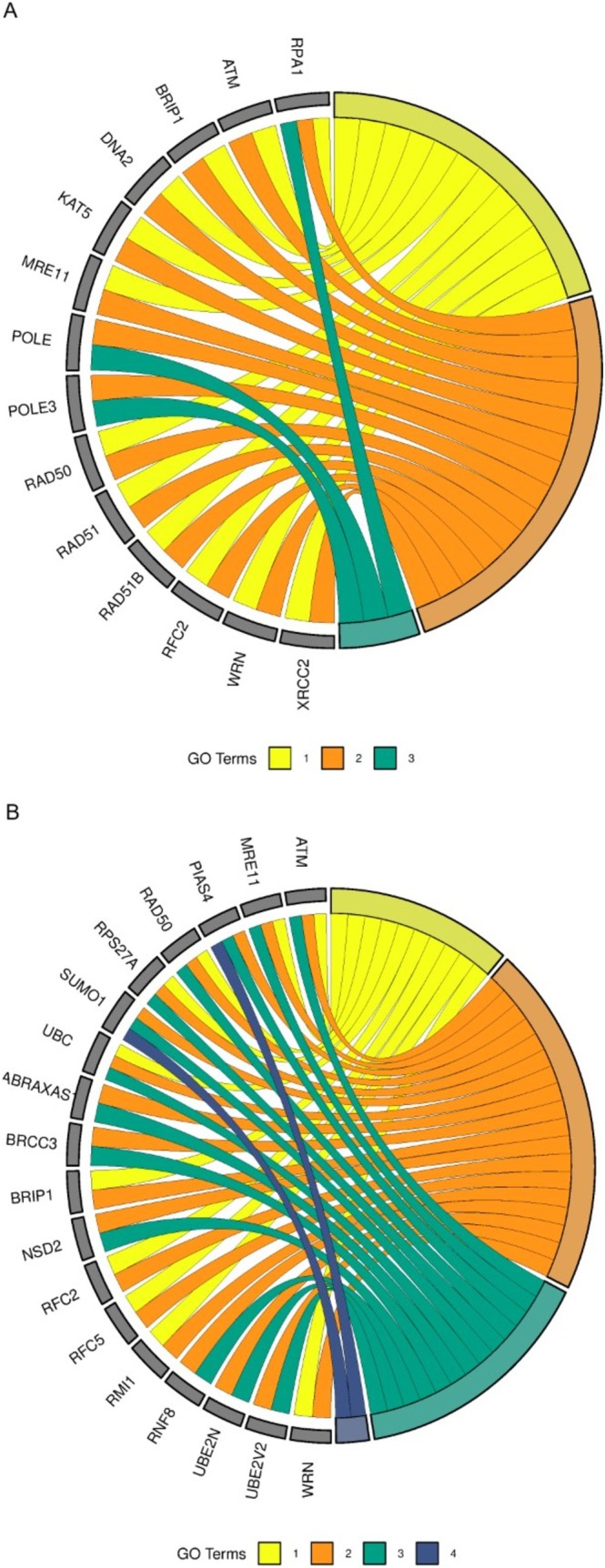
**Enriched replication pathways**. (A) Enriched replication pathways for ASD group: 1) presynaptic phase of homologous DNA pairing and strand exchange; 2) HDR through homologous recombination (HRR); 3) activation of the prereplicative complex. (B) Enriched replication pathways for the placenta group: 1) HDR through HRR; 2) Processing of DNA double‐strand break ends; 3) recruitment and ATM‐mediated phosphorylation of repair and signalling proteins at DNA double strand breaks; 4) SUMOylation of DNA replication proteins. GO = gene ontology. *P* values were corrected for multiple testing using the Benjamini–Hochberg false discovery rate (FDR), and pathways were considered significant at FDR‐adjusted *p* < 0.05.

**FIGURE 7:**
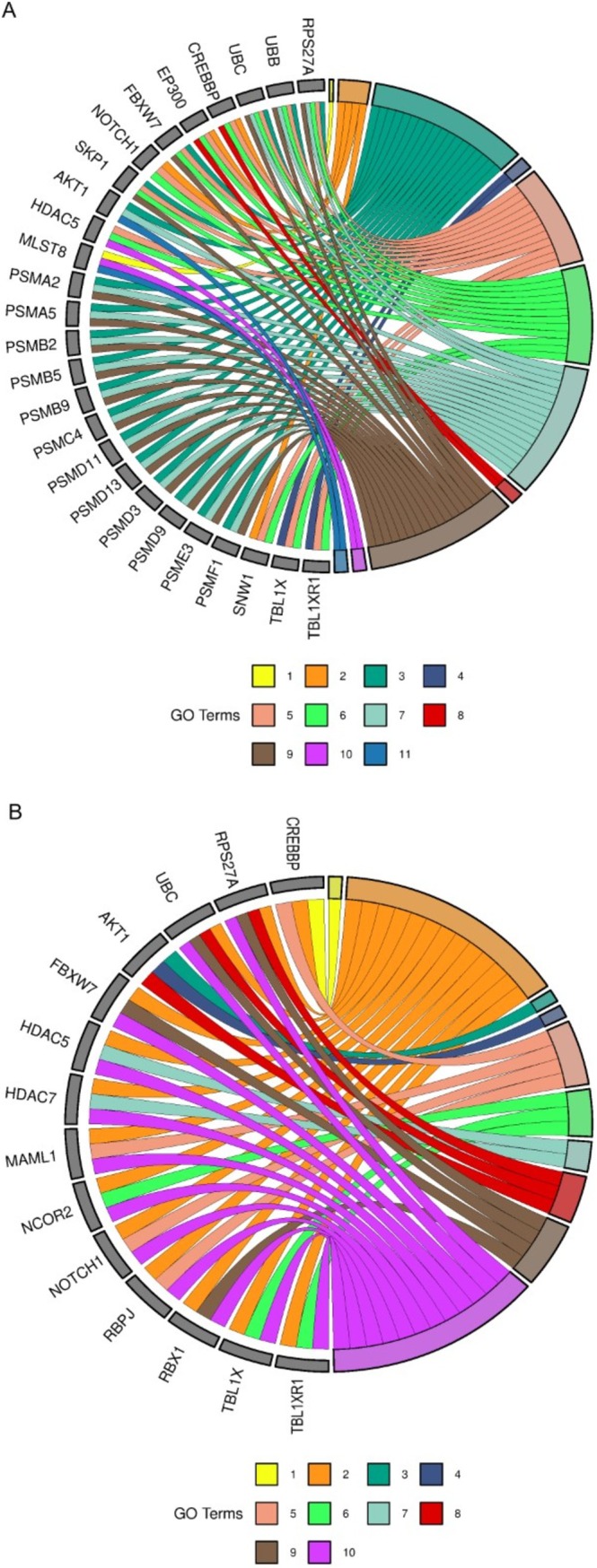
**Enriched ASD pathways**. (A) Enriched ASD pathways for the ASD group: 1) amino acids regulate mTORC1; 2) pre‐NOTCH transcription and translation; 3) negative regulation of NOTCH4 signalling; 4) regulation of MECP2 expression and activity; 5) constitutive signalling by NOTCH1 HD + PEST domain mutants; 6) NOTCH1 intracellular domain regulates transcription; 7) regulation of PTEN stability and activity; 8) activation of anterior HOX genes in hindbrain development during early embryogenesis; 9) regulation of RUNX2 expression and activity; 10) regulation of PTEN gene transcription; 11) PIP3 activates AKT signalling. (B) enriched ASD pathways for the placenta group: 1) activation of anterior HOX genes in hindbrain development during early embryogenesis; 2) constitutive signalling by NOTCH1 HD + PEST domain mutants; 3) PI5P, PP2A and IER3 Regulate PI3K/AKT signalling; 4) PIP3 activates AKT signalling; 5) pre‐NOTCH transcription and translation; 6) regulation of MECP2 expression and activity; 7) regulation of PTEN gene transcription; 8) regulation of PTEN stability and activity; 9) regulation of RUNX2 expression and activity; 10) NOTCH1 intracellular domain regulates transcription. GO = gene ontology. *P* values were corrected for multiple testing using the Benjamini–Hochberg false discovery rate (FDR), and pathways were considered significant at FDR‐adjusted *p* < 0.05.

To further refine the analysis, pathways were categorized into four groups: transcription, replication, immune system and ASD‐related pathways. Genes enriching the largest number of pathways within each category were identified. For transcription, immune system and ASD pathways, a gene had to enrich at least three pathways to be considered relevant, while for replication pathways, the cutoff was two pathways due to the smaller number of genes. The cutoff was determined following data insertion instructions presented on the *SRplot* platform. The immune system pathways are represented for ASD and placenta in Figure 4A and B, respectively. Figure 5A and B show the transcription pathways for ASD and placenta. The replication pathways are represented for ASD in Figure 6A and for placenta in Figure 6B. The ASD‐associated pathways are represented for ASD in Figure 7A and for placenta in Figure 7B.

### 3.4 SFARI Search for Overlapping Genes

**FIGURE 8:**
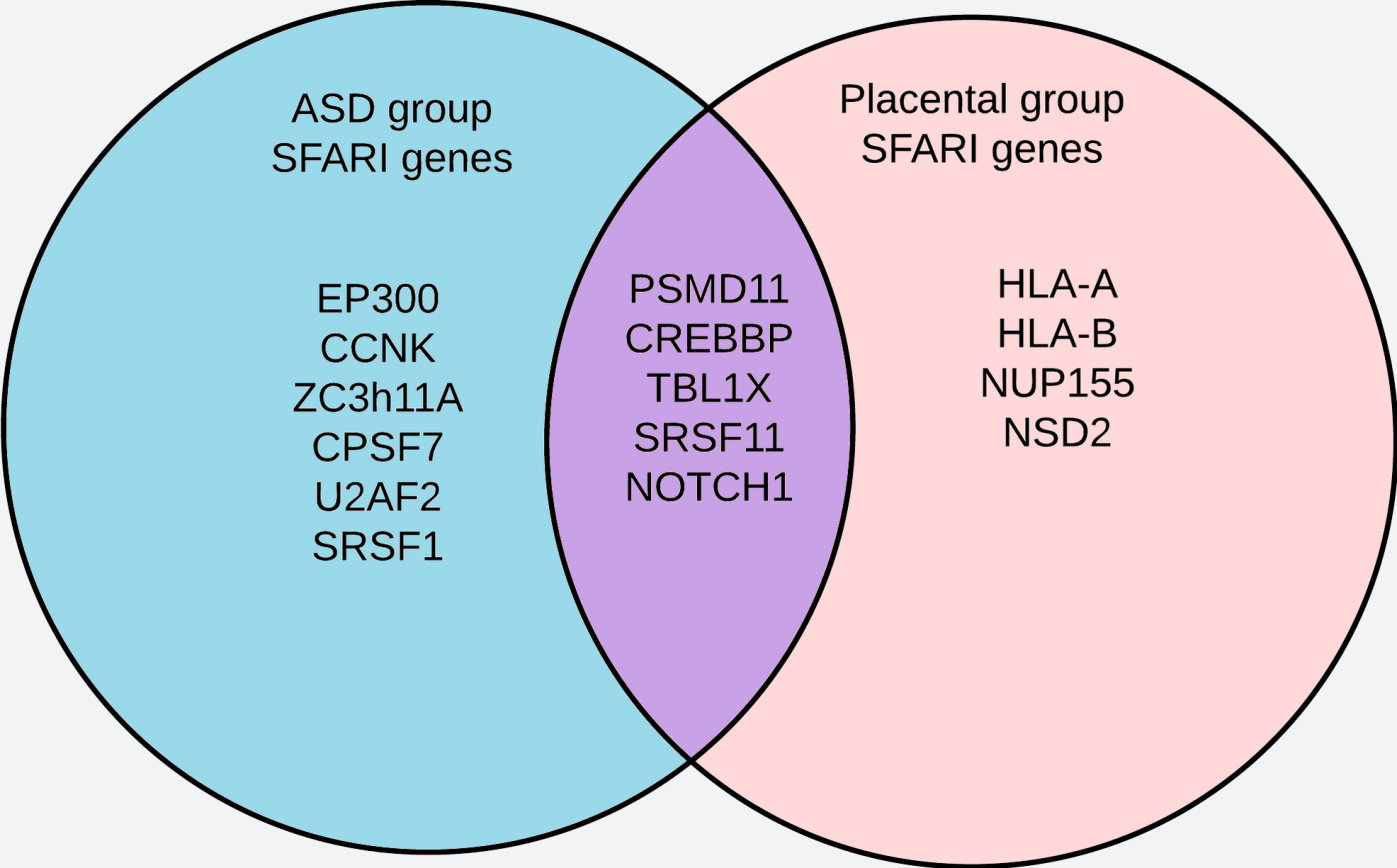
**Overlapping genes identified in the *SFARI* analysis**. Six genes were identified in the ASD group after the *SFARI* gene search, including *EP300*, *CCNK*, *ZC3H11A*, *CPSF7*, *U2AF2* and *SRSF11*. Four genes were identified in the placenta group, including *HLA‐A*, *HLA‐B*, *NUP155* and *NSD2*. Five genes were shared between ASD and placenta groups after the *SFARI* gene search, including *PSMD11*, *CREBBP*, *TBL1X*, *SRSF11* and *NOTCH1*.

Furthermore, a comparison between the ASD (Table 1) and placenta (Table 2) groups with the *SFARI* database was performed to identify overlapping genes associated with ASD risk. This analysis, summarized in the Venn diagram in Figure 8, revealed five genes shared between the two groups.

**TABLE 1:** circRNA–miRNA–mRNA‐SFARI network identified in the ASD group.

ASD circRNA	ASD miRNA	SFARI gene	Pathways
hsa‐CYP2U1_0001	miR‐4722‐5p	*PSMD11*	Immune system
hsa‐SFMBT2_0001	miR‐4722‐5p	*PSMD11*	Immune system
hsa‐ARID1A_0001	miR‐766‐5p	*PSMD11*	Immune system
hsa‐CYP2U1_0001	miR‐766‐5p	*PSMD11*	Immune system
hsa‐PAPPA_0016	miR‐766‐5p	*PSMD11*	Immune system
hsa‐KMT2E‐AS1_0001	miR‐766‐5p	*PSMD11*	Immune system
hsa‐SFMBT2_0001	miR‐766‐5p	*PSMD11*	Immune system
hsa‐ATP2C1_0001	miR‐766‐5p	*PSMD11*	Immune system
hsa‐PAPPA2_0005	miR‐942‐5p	*CREBBP*	ASD pathways
hsa‐CYP2U1_0001	miR‐942‐5p	*CREBBP*	ASD pathways
hsa‐ERC1_0001	miR‐942‐5p	*CREBBP*	ASD pathways
hsa‐PAPPA2_0005	miR‐6875‐3p	*TBL1X*	ASD pathways
hsa‐PAPPA_0016	miR‐6875‐3p	*TBL1X*	ASD pathways
hsa‐SCLT1_0001	miR‐6875‐3p	*TBL1X*	ASD pathways
hsa‐ERC1_0001	miR‐6875‐3p	*TBL1X*	ASD pathways
hsa‐PAPPA2_0005	miR‐4738‐3p	*SRSF11*	Transcription
hsa‐MAN1A2_0001	miR‐4738‐3p	*SRSF11*	Transcription
hsa‐PAPPA2_0005	miR‐129‐5p	*NOTCH1*	ASD pathways
hsa‐SCLT1_0001	miR‐129‐5p	*NOTCH1*	ASD pathways

**TABLE 2:** circRNA–miRNA–mRNA–SFARI network identified in the placenta group.

Placental circRNA	Placental miRNA	SFARI gene	Pathways
hsa‐MAN1A2_0008	miR‐4722‐5p	*PSMD11*	Immune system
hsa‐ZKSCAN1_0001	miR‐4722‐5p	*PSMD11*	Immune system
hsa‐MAN1A2_0008	miR‐942‐5p	*CREBBP*	ASD pathways
hsa‐MAN1A2_0003	miR‐942‐5p	*CREBBP*	ASD pathways
hsa‐MAN1A2_0002	miR‐942‐5p	*CREBBP*	ASD pathways
hsa‐FBXW7_0005	miR‐942‐5p	*CREBBP*	ASD pathways
hsa‐ZNF609_0001	miR‐942‐5p	*CREBBP*	ASD pathways
hsa‐CDYL_0005	miR‐942‐5p	*CREBBP*	ASD pathways
hsa‐HIPK3_0001	miR‐942‐5p	*CREBBP*	ASD pathways
hsa‐MAN1A2_0008	miR‐6875‐3p	*TBL1X*	ASD pathways
hsa‐MAN1A2_0003	miR‐6875‐3p	*TBL1X*	ASD pathways
hsa‐MAN1A2_0002	miR‐6875‐3p	*TBL1X*	ASD pathways
hsa‐FBXW7_0005	miR‐6875‐3p	*TBL1X*	ASD pathways
hsa‐ZNF609_0001	miR‐6875‐3p	*TBL1X*	ASD pathways
hsa‐CDYL_0005	miR‐6875‐3p	*TBL1X*	ASD pathways
hsa‐MAN1A2_0008	miR‐6832‐3p	*SRSF11*	Transcription
hsa‐MAN1A2_0003	miR‐6832‐3p	*SRSF11*	Transcription
hsa‐MAN1A2_0002	miR‐6832‐3p	*SRSF11*	Transcription
hsa‐CDYL_0005	miR‐6832‐3p	*SRSF11*	Transcription
hsa‐MAN1A2_0008	mir‐623	*NOTCH1*	ASD pathways
hsa‐MAN1A2_0003	mir‐623	*NOTCH1*	ASD pathways
hsa‐MAN1A2_0002	mir‐623	*NOTCH1*	ASD pathways
hsa‐FBXW7_0005	mir‐623	*NOTCH1*	ASD pathways
hsa‐ZNF609_0001	mir‐623	*NOTCH1*	ASD pathways

### 3.5 circRNA–miRNA–mRNA Interaction Network

**FIGURE 9:**
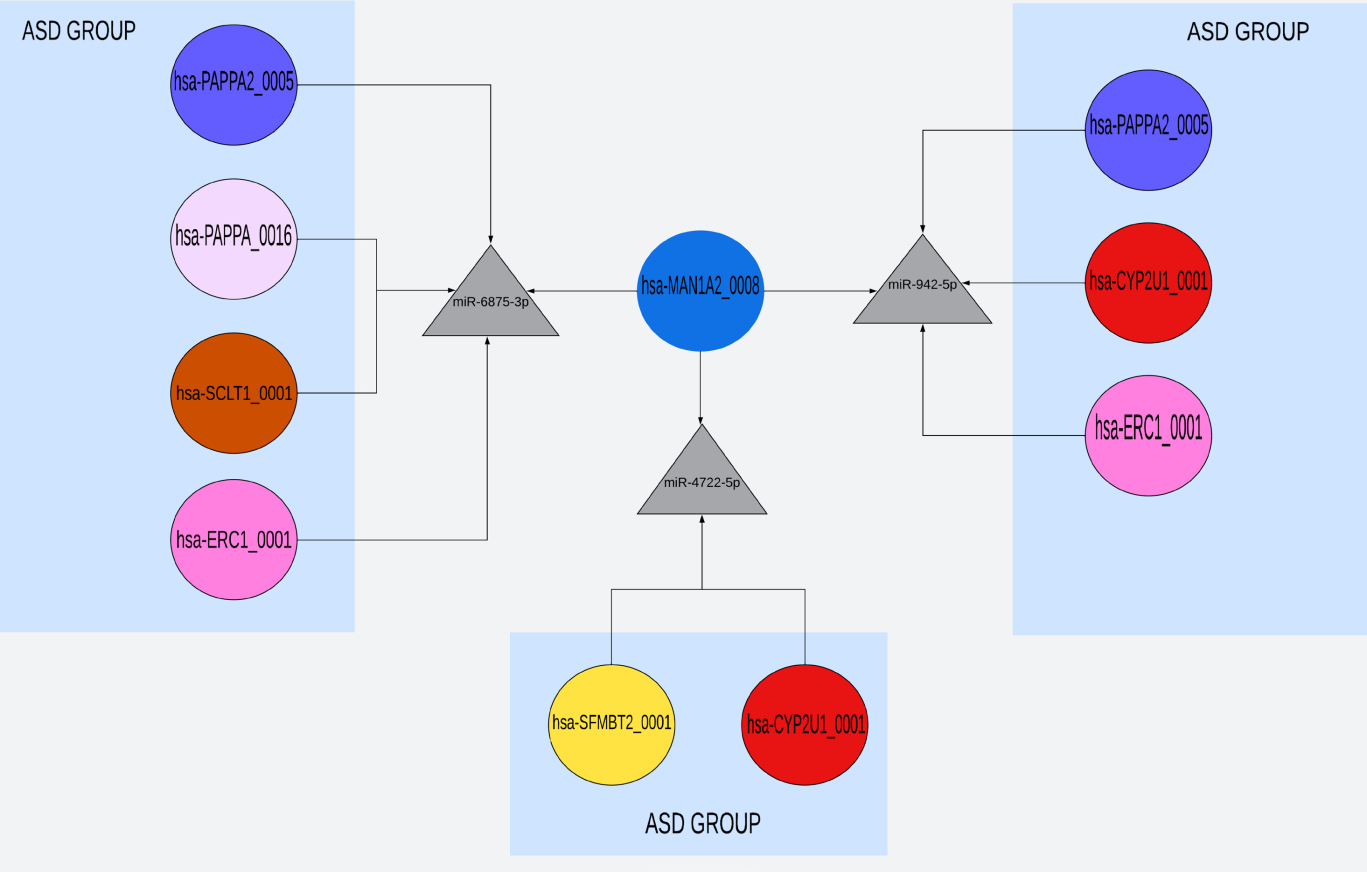
**Correlation between the circRNA hsa‐MAN1A2_0008 (placental group) and miRNA shared with circRNA from the ASD group**. The circRNA hsa‐MAN1A2_0008, identified in the placental group, targets the miRNA miR‐6875‐3p, miR‐4722‐5p and miR‐942‐5p, which are also targeted by circRNA associated with the ASD group. The miR‐6875‐3p is regulated by the circRNA hsa‐PAPPA2_0005, hsa‐PAPPA_0016, hsa‐SCLT1_0001 and hsa‐ERC1_0001 from the ASD group. miR‐4722‐5p is regulated by hsa‐SFMBT2_0001 and hsa‐CYP2U1_0001, while miR‐942‐5p is regulated by hsa‐PAPPA2_0005, hsa‐CYP2U1_0001 and hsa‐ERC1_0001.

**FIGURE 10:**
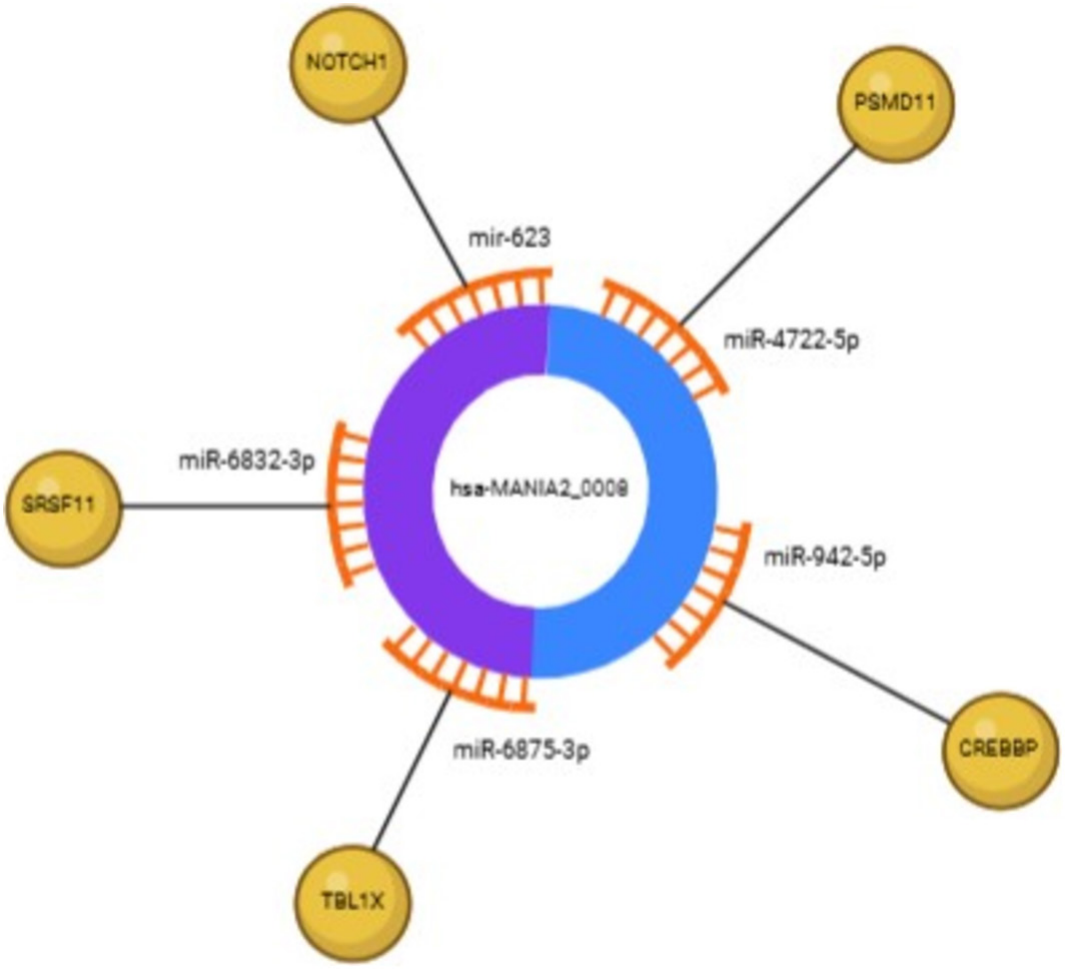
**circRNA–miRNA–mRNA interaction.** The circRNA hsa‐MAN1A2_0008 interacts with miR‐6832‐3p, miR‐623, miR‐4722‐5p, miR‐942‐5p and miR‐6875‐3p, probably affecting the expression of the targets *CREBBP*, *TBLX1*, *SRSF11*, *PSMD11* and *NOTCH1*.

Finally, an interaction network was constructed to explore the relationships among circRNA, miRNA and the overlapping genes identified in Figure 8. Through this analysis, we identified the circRNA hsa‐MAN1A2_0008 as a promising candidate, as it targets miR‐9832‐3p, miR‐623, miR‐4722‐5p, miR‐942‐5p and miR‐6875‐3p that regulate all five overlapping genes (*CREBBP*, *TBL1X1*, *SRSF11*, *PSMD11* and *NOTCH1*). Notably, three of these miRNA (miR‐6875‐3p, miR‐942‐5p and miR‐4722‐5p) were also shared with the ASD group, as shown in Figure 9. The interaction network is illustrated in Figure 10, highlighting the potential of hsa‐MAN1A2_0008 as a key target for future research into the role of the placenta in ASD. The miRNA described in the hsa‐MAN1A2_0008 interaction has different profiles of placental expression, as described in Tables S1 and S2.

## 4 Discussion

The presence of differentially expressed circRNA in ASD has been previously reported (Ghafouri‐Fard et al. 2022), however, their relevance to the disorder remains unclear. Our work explores this gap by investigating pathways affected by the most frequently differentially expressed circRNA identified in ASD and circRNA expressed in placenta, identifying correlations between pathways and affected miRNA, suggesting a promising highly expressed circRNA in the placenta, which may be able to interact with genes relevant to ASD outcome.

When it comes to research targeting circRNA, it is important to keep in mind the stoichiometric ratio of circRNA to miRNA molecules within the cell. There is a difference between the number of copies of each molecule, as the circRNA is produced at less than 2.5 copies per cell, whereas miRNA are often produced at 90–80,000 copies per cell. This disparity emphasizes the need to focus on highly expressed circRNA in target tissues to optimize their function as miRNA sponges (Arthurs et al. 2022).

The search for the most relevant circRNA in the context of ASD revealed important pathways potentially affected by these molecules. More specifically, biological pathways associated with the immune system, transcription and replication were elicited, which is expected for ASD. Several articles already highlighted the neuroimmune background associated with ASD, including increased levels of inflammatory cytokines such as IL‐1 and IL‐6 in the serum and in the brain (Than et al. 2023). Regarding the regulation of transcription, one example comes from the VPA model of ASD: if an analogue of VPA called valpromide—which does not have the histone deacetylase inhibition characteristic—is administered in the same way as VPA, the offspring do not present ASD‐like features, demonstrating that the processes involving transcription regulation are extremely important for the outcome (Ko et al. 2018). Finally, proliferation alterations were already described in ASD. For example, an increase in the cellularity of brain regions such as the prefrontal cortex (Varghese et al. 2017) in humans and the medial prefrontal cortex in the VPA model (Codagnone et al. 2015).

Besides that, we also demonstrated that the circRNA relevant to ASD also affects pathways that are classically involved in ASD pathophysiology such as Notch and AKT signalling pathways. The Notch pathway is highly activated in the BTBR animal model of autism, modulating T cell activity (Yao et al. 2022), playing important roles in the remodelling of synapses (Zhang et al. 2023) and in the activity of DNA methyltransferases (Li et al. 2023). AKT, in turn, is a protein largely associated with the PTEN/mTOR pathway, which was described as altered in several ASD models and human studies, leading to alterations in multiple cellular processes like synaptic pruning (Xiaoyan et al. 2024). The activation of the PI3K/AKT pathway was already associated with improvements in social behaviour deficits, repetitive stereotyped and compulsive behaviours, anxiety and exploratory behaviour in animal models of ASD.

The placenta is a major interface between mother and embryo/foetus, providing an environment of interchange of nutrients, signalling molecules, immune system molecules, among others. Several alterations in the placenta were already described in ASD, such as malperfusion, due to morphological alterations and methylation changes (Straughen et al. 2017; Bahado‐Singh et al. 2021). By focusing on the circRNA highly expressed in the placenta, we identified pathways involving replication, transcription, the immune system and also pathways relevant to ASD, demonstrating this possible interaction (the main pathways were displayed on Figures 4, 5, 6, 7).

When we analyzed the genes associated with these biological pathways in both ASD and placenta groups and submitted them to the *SFARI* database, we identified five overlapping genes (*PSMD11*, *CREBBP*, *TBL1X*, *NOTCH1* and *SRSF11*) between the ASD and placenta groups (Figure 8). These genes serve as key targets for a proposed circRNA–miRNA–mRNA regulatory network. Their functions span immune regulation (*PSMD11*), transcription (*SRSF11*) and pathways critical to ASD development (*CREBBP*, *TBL1X* and *NOTCH1*).

To assemble a network, we investigated the miRNA targets of the overlapping genes and the circRNA that target these miRNA. We discovered a highly expressed circRNA in placenta (has_MAN1A2_0008) that targets miRNA that regulates all five overlapping genes, as shown in Table 1. The miR‐4722‐5P targets the *PSMD11* gene, which is correlated with immune system pathways; the miR‐942‐5p, miR‐6875‐3p and miR‐623 regulate genes associated with pathways relevant to ASD development (*CREBBP*, *TBL1X* and *NOTCH1*, respectively) and the miR‐6832‐3p targets the *SRSFF11* gene, which is associated with transcription pathways. All miRNAs in this network are expressed in the placenta, showing variable expression levels (Table S1; miRNATissueAtlas 2025) (Rishik et al. 2025). Interestingly, three of the five miRNA (hsa‐miR‐623, hsa‐miR‐4722‐5p and hsa‐miR‐942‐5p) have already been described as altered in placental conditions, including selective intrauterine growth restriction, preeclampsia (PE) and gestational diabetes mellitus; besides that, interactions with circRNAs were also observed (Wen et al. 2017; Wang et al. 2019; Liu et al. 2022; Yu et al. 2023; Tian et al. 2024; Liao et al. 2024; Chu et al. 2024; Guo et al. 2025; Wu and Zhao 2025). The question regarding the impact of low or high expressed miRNA in a specific tissue is very complex, especially when taking circRNA into account. Even when the tissue‐level expression of a miRNA is low, its function can be critical within the placental microenvironment. In this scenario, susceptibility to sequestration by highly expressed circRNAs may be relatively greater, which justifies including such miRNAs in the network analysis. On the other hand, highly expressed miRNA usually indicates relevant roles in the respective tissue. This discussion is complex and is not exhausted by the proposed analyses.

The five genes associated with the network, as shown in Figure 10, are involved in processes essential for placental development, particularly targeting trophoblastic cell proliferation and angiogenesis, which are crucial during early placental formation across various species (Hemberger et al. 2020). Besides that, the impact of these key processes on placental development has already been correlated with ASD aetiology (Chang et al. 2017; Kliman et al. 2021; Walker et al. 2013).

Among the genes studied, *TBL1X*, *CREBBP* and *NOTCH1* are associated with ASD pathways and also play a significant role in trophoblastic cell differentiation and migration (Lackner et al. 2023; van Voorden et al. 2023) in addition to being considered potential targets in the pathogenesis of PE (Sadeghi et al. 2021). Studies in mouse models demonstrate that genetic ablation of *TBL1X* leads to a trophoblast stem cell differentiation deficit, impairing placental development (Lackner et al. 2023). Furthermore, *TBL1X* regulates *MECP2* expression and activity, a major transcriptional regulator of the hypothalamus–pituitary–adrenal axis targeting other genes critical for placental processes, such as *BDNF* and *TH* (associated with trophoblast growth and PE) and *SGK1* (linked to embryonic implantation errors) (Meakin et al. 2018).

The *CREBBP* gene expression was analyzed across 100 placental biopsies from PE patients and healthy pregnant women using RT‐qPCR, and it showed that the *CREBBP* gene expression was higher in PE patients, suggesting a role for *CREBBP* in the pathogenesis of PE due to its crucial role in angiogenesis and hypoxia (Sadeghi et al. 2021). The *NOTCH1* gene expression has already been reported in a multiple number of human placental trophoblasts which might suggest an involvement of the Notch pathway in the trophoblast differentiation program, and its contribution to key processes in placental development. NOTCH protein expression in placental blood vessels has also been reported, implying a possible contribution of the Notch pathway in placental angiogenesis (Zhao and Lin 2012). Besides that, it was also demonstrated that NOTCH proteins were also decreased in preeclamptic placentas compared to controls and, therefore, might be associated with the onset of PE (Cobellis et al. 2007). Notably, *TBL1X*, *CREBBP* and *NOTCH1* are also part of the Notch signalling pathway.

Subsequently, the PSMD11 gene is involved with immune system pathways, affecting mainly NFKB and interleukin‐1 signalling. Many studies have already described the relevance of its molecules to trophoblastic cells (Armistead et al. 2020; Nestler 1993) and its roles in preeclamptic placentas (Armistead et al. 2020). Lastly, SRSF11 contributes to transcription factor (TF) pathways that are critical for trophoblast development (Papuchova and Latos 2022) and has been implicated in ASD aetiology (Santos‐Terra et al. 2021). Its involvement highlights the shared molecular mechanisms underlying placental and neurodevelopmental processes.

It is also important to highlight, as shown in Figure 9, that the miRNA targeted by hsa‐MAN1A2_0008 is also shared with circRNA present in the ASD group, which targets the same genes and pathways, reinforcing its relevance to ASD aetiology. Specifically, miR‐6875‐3p is regulated by the circRNA hsa‐PAPPA2_0005, hsa‐PAPPA_0016, hsa‐SCLT1_0001 and hsa‐ERC1_0001 from the ASD group; miR‐4722‐5p by hsa‐SFMBT2_0001 and hsa‐CYP2U1_0001; and miR‐942‐5p by hsa‐PAPPA2_0005, hsa‐CYP2U1_0001 and hsa‐ERC1_0001. This shared regulatory network emphasizes the circRNA hsa‐MAN1A2_0008 as a promising target for ASD aetiology research.

Our findings suggest that placental circRNA may play an important role in ASD aetiology. Given that ASD is a neurodevelopmental disorder, placental alterations could significantly influence its development. The circRNA–miRNA–mRNA interactions identified in this study provide new insights into how these molecules might be related to placental alteration and contribute to ASD aetiology.

## 5 Limitations

The current work performed a series of bioinformatic analyses based on literature search and data provided by several software already validated and commonly used in this field. However, no experimental validation (in vitro or in vivo) was performed, posing an important limitation and also a perspective for further research. Besides that, challenges inherent in circRNA studies such as nomenclature inconsistencies and lack of standardization throughout databases also limit the analyses carried out in this study.

## Ethics Statement

This study did not require ethical approval as it was based solely on publicly available data sources. No human participants, personal data or animal subjects were involved in the research.

## Consent

Not applicable. The study did not involve human participants or primary data collection; all data analyzed were from publicly available sources.

## Conflicts of Interest

The authors declare no conflicts of interest.

## Supporting information


**Table S1:** Placental Expression of miRNA targeted by circRNA hsa‐MAN1A2_0008 according to miRNA Tissue Atlas 2025 (Rishik et al. 2025) (expressed as reads per million mapped).
**Table S2**: Placental Expression of miRNA targeted by circRNA hsa‐MAN1A2_0008 according to literature research.

**Table S3:** 72 pathways identified in the ASD group.

**Table S4:** 70 pathways identified in the Placenta group.

## Data Availability

The data that support the findings of this study are available from the corresponding author upon reasonable request.
