# Supplementary material for: Placental Alterations in Autism Spectrum Disorder: An In Silico Approach to circRNA–miRNA–mRNA Networks
Source: Int J Dev Neurosci. 2025 Nov 11;85(7):e70064. doi: 10.1002/jdn.70064 (PMC12611451; doi:10.1002/jdn.70064)
Supplement: Supplementary file 1 — Table S1: Placental Expression of miRNA targeted by circRNA hsa‐MAN1A2_0008 according to miRNA Tissue Atlas 2025 (Rishik et al. 2025) (expressed as reads per million mapped). Table S2: Placental Expression of miRNA targeted by circRNA hsa‐MAN1A2_0008 according to literature research. [file JDN-85-0-s003.docx]

**Supplementary Table 1**: Placental Expression of miRNA targeted by circRNA hsa-MAN1A2_0008 according to miRNA Tissue Atlas 2025 [(Rishik et al., 2025)](https://www.zotero.org/google-docs/?oIOuyj) (expressed as reads per million mapped).

| **miRNA** | **Average Expression** | **Standard Deviation** |
| --- | --- | --- |
| hsa-miR-623 | 0.261 | 0.5184 |
| hsa-miR-4722-5p | 19.2263 | 29.8885 |
| hsa-miR-942-5p | 4.5251 | 5.2625 |
| hsa-miR-6875-3p | 0.0038 | 0.0204 |
| hsa-miR-6832-3p | 0.1542 | 0.2812 |

**Supplementary Table 2**: Placental Expression of miRNA targeted by circRNA hsa-MAN1A2_0008 according to literature research.

| **miRNA** | **Results** | **Technique** | **Context** | **Study** |
| --- | --- | --- | --- | --- |
| hsa-miR-623 | Downregulated | Microarray + RT-qPCR | Selective intrauterine growth restriction | [(Wen et al., 2017)](https://www.zotero.org/google-docs/?3j0ci3) |
| hsa-miR-4722-5p | Description of interaction with circRNA_0395, no direct results about differential expression | Bioinformatics | Gestational Diabetes Mellitus | [(Wang et al., 2019)](https://www.zotero.org/google-docs/?0JTdYg) |
| hsa-miR-942-5p | Downregulated; description of interaction with circ_0085296 | RT-qPCR | Preeclampsia | [(Liu et al., 2022)](https://www.zotero.org/google-docs/?xHRMPA) |
|  | Downregulated; description of interaction with circ_CRIM1 | RT-qPCR | Preeclampsia | [(Yu et al., 2023)](https://www.zotero.org/google-docs/?JOoCr9) |
|  | Downregulated; description of interaction with circ_0030042 | RT-qPCR | Preeclampsia | [(Tian et al., 2024)](https://www.zotero.org/google-docs/?IlPlNO) |
|  | Downregulated; description of interaction with circ_PAPPA2 | RT-qPCR | Preeclampsia | [(Liao et al., 2024)](https://www.zotero.org/google-docs/?1Bl0mA) |
|  | Donwregulated | RT-qPCR | Gestational Diabetes Mellitus | [(Chu et al., 2024)](https://www.zotero.org/google-docs/?MyNPLk) |
|  | Downregulated; description of interaction with circ_0008440 | RT-qPCR | Preeclampsia | [(Guo et al., 2025)](https://www.zotero.org/google-docs/?NFwhFL) |
|  | Downregulated; description of interaction with circ_0015382 | RT-qPCR | Preeclampsia | [(Wu & Zhao, 2025)](https://www.zotero.org/google-docs/?kl9tiQ) |
| hsa-miR-6875-3p | - | - | - | - |
| hsa-miR-6832-3p | - | - | - | - |
